# Perceptions of commercial snack food and beverages for infant and young child feeding: A mixed‐methods study among caregivers in Kathmandu Valley, Nepal

**DOI:** 10.1111/mcn.12711

**Published:** 2019-06-21

**Authors:** Nisha Sharma, Elaine L. Ferguson, Atul Upadhyay, Elizabeth Zehner, Suzanne Filteau, Alissa M. Pries

**Affiliations:** ^1^ Helen Keller International New York New York; ^2^ Department of Population Health, Faculty of Epidemiology and Population Health London School of Hygiene and Tropical Medicine London UK

**Keywords:** double burden, complementary feeding, complementary foods, Nepal, nutrition, snack

## Abstract

Ensuring nutritious complementary feeding is vital for child nutrition. Prior research in Kathmandu Valley found high consumption rates of commercially produced snack foods among young children, which are often energy‐dense/nutrient poor. This mixed‐methods study was conducted to elicit Nepali caregivers' perceptions of commercial snack foods and beverages and factors influencing their use for young child feeding. Seven facilitated focus group discussions (FGD) were conducted with Kathmandu Valley caregivers of children 12–23 months, and a survey of 745 primary caregivers of children 12–23 months of age was then conducted. During the FGD, caregivers reported commonly providing commercial food and beverage products to their children as snacks, and 98.6% of caregivers participating in the survey reported feeding their child such a food in the previous week. Because of processing and packaging, snack foods were not trusted by many FGD participants and considered as “junk foods” and not healthy for children. However, commercial snack foods were consistently ranked highly on convenience, both because of minimal preparation and ease of feeding; 48.5% of all surveyed caregivers reported providing a snack food because of convenience. Other family members' diets or provision of snack foods as treats also influenced children's consumption of these snack foods and beverages. This study indicates that caregivers of young children prefer snack options that are nutrient rich; however, this may conflict with preferences for foods that require minimal preparation and are appealing to young children. Such findings carry programmatic implications for interventions aiming to address children's diet quality in urban Nepal.

**Key messages**

- Primary caregivers of children 12–23 months of age commonly feed commercial snack foods and beverages in Kathmandu Valley.
- Despite the perception that many of these foods are not healthy, caregivers reported using them because of child preference, ease of preparation, and ease of feeding.
- Caregivers also noted children modelling eating practices of older children/relatives, and the provision of commercial snack food and beverage products as gifts/treats by adults was common.
- In order to tackle the growing use of unhealthy snack foods and beverages for young child feeding, the range of factors and perceptions influencing caregiver behaviours need to be understood and addressed.

## 1 INTRODUCTION

A “nutrition transition” has been identified in many low‐ and middle‐income countries (LMIC), with diets increasing in added sugars, fats, and refined carbohydrates (Popkin, Adair, & Ng, 2012). Among children living in countries experiencing such dietary transitions, there has been marked growth in consumption of processed foods, including commercially produced snack food and beverage products (Faber & Benade, 2007; Huffman, Piwoz, Vosti, & Dewey, 2014; Pantoja‐Mendoza, Meléndez, Guevara‐Cruz, & Serralde‐Zúñiga, 2015; Pries, Huffman, Mengkheang et al., 2016; Woo et al., 2013).

Despite improvements in the nutritional status of children in Nepal, stunting among children under 5 years of age remains high at 36% and wasting at 10% nationally and 32% and 9% in urban areas (MOHP New ERA and ICF DHS Program, 2017). Snack food consumption is prevalent among young children in Nepal, particularly in urban areas (Huffman et al., 2014). A survey among Kathmandu Valley children 6–23 months of age found that 57% and 43% had consumed commercially produced biscuits/cookies and sweets/candy, respectively, in the day prior to interview, and the proportion of children consuming snack foods was higher than those consuming dark green leafy vegetables (35%), orange‐fleshed fruits (1%) and vegetables (8%), or eggs (24%) (Pries, Huffman, Adhikary et al., 2016).

These consumption patterns are cause for concern; often, snack food products are energy‐dense and micronutrient‐poor (Lucan, Karpyn, & Sherman, 2010; Monteiro, Levy, Claro, de Castro, & Cannon, 2011; Sekiyama, Roosita, & Ohtsuka, 2012; Waseem et al., 2014). Exposure to foods early in life has also been shown to establish dietary preferences that remain throughout childhood and into adulthood (Birch & Doub, 2014; Mennella, 2014), potentially establishing unhealthy eating patterns and increasing risk of overnutrition and related chronic disease later in life. Over one quarter of women in urban Nepal are overweight/obese (MOHP New ERA and ICF DHS Program, 2017), and overnutrition affects 40 million children globally, with the majority of these children living in LMIC (World Health Organization, 2011). In a context such as urban Nepal, understanding the drivers behind caregivers' decisions to use commercial snack foods and beverages for young child feeding could lead to insights on how to mitigate increasing consumption rates and to prevent overweight/obesity in adulthood. This study assessed a sample of Kathmandu Valley caregivers' perceptions of commercial snack foods and beverages and elucidated factors that influence their use for child feeding.

## 2 METHODS

A mixed‐methods design was used for this study; a qualitative component involved focus group discussions (FGD) and participatory exercises, and a quantitative component involved a structured interview during a survey. The qualitative component was conducted first in November 2016 in order to inform survey tool design and methods, which was then conducted February–April 2017.

### 2.1 FGDs and participatory exercises

Seven facilitated group discussions, including participatory exercises, among caregivers of children 12–23 months of age were conducted to elicit caregivers' perceptions of commercial snack foods and beverages and factors influencing their use for infant and young child feeding. Thirty‐two caregivers of children 12–23 months of age were purposively sampled from areas of Kathmandu Valley anticipated to have populations of varying socio‐economic status (SES). Discussion groups were stratified by three caregiver types: (a) non‐working mothers, (b) working (paid employment) mothers, and (c) grandmothers. Participants were purposively recruited with the assistance of female community health volunteers in each location who were able to identify caregivers with a child 12–23 months of age.

#### 2.1.1 Tool and process for group discussions and exercises

Group discussions were led by a facilitator and were conducted in a private space and audio recorded. The process began with a discussion on child feeding, covering the foods fed to children and caregivers' considerations around these food choices. This led into a guided discussion on *khaja* (the Nepali word for “snack”) and caregivers' definitions of snacks and snacking, followed by a series of guided participatory exercises based on behaviour‐centred design methods (Aunger, White, Greenland, & Curtis, 2017), including: free‐listing of all foods fed to young children as snacks, categorization of foods into groups based on perceived similarities, and ranking based on continuums of health, child preference, convenience, and cost. A discussion guide was used by the facilitator; this tool and the methods were pre‐tested prior to data collection to ensure participant understanding.

The audio‐recorded group discussions were transcribed verbatim and translated from Nepali to English. Each transcript was checked against the recording to ensure completeness and accuracy of translation. NVivo 11 was used for the analysis of qualitative data. A data‐driven inductive thematic analysis was conducted following methods outlined by Braun and Clarke (2006), which has been used as a basis for analysis in similar studies seeking to understand caregivers' feeding practices for young children (Jacquier, Gatrell, & Bingley, 2016). Transcripts were initially auto‐coded on the basis of the questions in the discussion guideline; these were reviewed, and three major domains specific to commercial snack foods were identified: (a) the use of commercial snack foods and beverages for children, (b) reasons for feeding commercial snack food to the children, and (c) caregiver's perception of commercial snack foods for young children. Within domains, emerging themes were identified and coded; for example, for the domain “reasons for feeding commercial snack food to children”, eight themes were identified, and under the domain “caregiver's perceptions of commercial snack foods for young children”, four themes were identified. The process of coding was led by one researcher on the team and reviewed by another researcher. As the coded data were grouped thematically, a consensus was developed between the two researchers on the themes generated.

### 2.2 Quantitative survey

A cross‐sectional representative survey was conducted among 745 primary caregivers of children 12–23 months of age living in Kathmandu Valley. Primary caregivers were defined as caregivers who typically provided the majority of care to the child in a day. A multistage cluster random sampling procedure was used for this survey. Sampling units were based on municipality wards; using ward population estimates from the 2011 Nepal Census (CBS, 2012), 78 clusters of nine child–caregiver pairs were assigned to these units based on probability proportional to size (PPS). The final 78 clusters were assigned across 68 municipality wards: 8 in Bhaktapur, 42 in Kathmandu, and 18 in Lalitpur. Participants in each cluster were sampled 2–3 days prior to scheduled data collection by a trained recruitment team through door‐to‐door visits for random selection. Caregivers were excluded from participation (a) if the child was severely ill; (b) if the child/caregiver did not live in Kathmandu Valley; and (c) if the child had a congenital/physical malformation that inhibited feeding.

#### 2.2.1 Questionnaire design and data management

Data were collected on demographic and socio‐economic characteristics pertaining to the caregiver, household, and child. Children's frequency of consumption of snack foods and beverages in the week prior to interview were also measured using methods adapted from Faber and Benade (2007) and which have been previously used in this Nepali context (Pries, Huffman, Adhikary, et al., 2016). A module regarding caregivers' decisions to feed snack foods and sugar‐sweetened beverages to their child was developed based on preliminary FGD findings. Caregivers were asked to provide open‐ended responses on reasons why they chose to feed specific types of snack foods and beverages to their child in the previous week; responses were captured with pre‐coded response options based on themes identified in the FGD, and any additional non‐pre‐coded responses were entered as text. All tools for this survey were translated into Nepali, back‐translated, and pre‐tested prior to data collection. Data were cleaned and open‐response entries were translated from Nepali to English. Reported reasons for providing snack food and beverages to children were coded based on finalized themes emerging from the FGD findings. Wealth quintiles were developed on the basis of principal component analysis using variables related to caregiver SES (Vyas & Kumaranayake, 2006). Descriptive statistics and Pearson's χ^2^ tests were run using Stata 15.

Ethical approval for this study was obtained from the London School of Hygiene and Tropical Medicine and the Nepal Health Research Council. Written informed consent was obtained from all FGD and quantitative survey participants.

## 3 RESULTS

Findings from the qualitative component of this study are first presented; the three identified domains and their themes are detailed below. Findings from the quantitative survey regarding reasons for the use of commercial snack foods/beverages are then presented.

### 3.1 Qualitative FGDs

1. The use of commercial snack foods and beverages for children

Commercially packaged foods, including beverages such as juice drinks and chocolate/malt powder‐based drinks and foods such as instant noodles, candies, chocolates, savoury snacks, and biscuits, were consistently mentioned as *khaja* (Nepali word for “snack”) for children across all groups, in addition to home‐made foods such as *jaulo* (porridge made of rice and lentil), milk, *lito* (porridge made of grains and legumes), *dal bhat* (rice, lentil, vegetables, pickle, and meat/fish), boiled eggs, and fruits. For both commercial and non‐commercial foods, several caregivers reported minimal differences in foods fed as *khana* (Nepali word for the “main meals”) and *khaja* for young children; *lito*, *jaulo*, and *dal bhat* were given as both meals and snacks to young children. Caregivers noted that foods fed as *khaja* began to differ as children grew up, with one difference being the introduction of commercial foods. Home‐made foods were more suitable for young children as these foods were soft, whereas commercial snack foods, such as instant noodles, were harder and considered more appropriate for older children: “While they are young they eat home‐made food, they may like market foods when they grow up” (Mother, mid/high SES).

- Reasons for feeding commercial snack foods to young children

Factors influencing caregivers' decisions to use commercial food products for young child feeding were voiced throughout FGDs. These reasons are detailed here:

- Child likes it: All caregivers reported being motivated by a child's food preferences and reported opting to feed commercial snack food and beverages because these foods were most liked by children: “(He) eats biscuit, I give (him) whatever he prefers” (Grandmother, mid SES).
- Lack of time: Caregivers reported using commercial snack foods when they did not have time to prepare home‐made foods. This occurred particularly when caregivers were rushing to go somewhere or when they were working (inside or outside the home). Commercial foods, such as biscuits, were easy for them to give to their children at such busy times*:* “When I can't make time, I give such foods.” (Mother, working, mid/high SES)/“Sometimes I do not have time to prepare and give food due to household work, so I give that for convenience” (Mother, low SES).
- Other adults provide as a gift/treat: Grandmothers in particular reported that they do not often provide commercial snack foods to their child, but rather other household members, neighbours, or visitors/guests provide them: “I try to avoid it (cheeseballs) as much as possible but sometimes others buy it for the child.” (Grandmother, mid SES) /“Child happily accepts it, they (visitors) wonder what should they buy for the child and then buy a packet of cheese balls for one hand and a chocolate for another” (Grandmother, mid SES).
- Child demand: Caregivers reported that children demand or cry for commercial snack foods: “Sometimes (he) quarrels and asks and I give (cheeseballs and noodles), sometimes juice drinks” (Mother, low SES). Several caregivers reported these demands often occurred when they were outside the home: “It is a problem when I am out with my child. (He) will not move a step unless I buy it and at the end I have to buy. My child sees those things hanging in the shops” (Mother, working, mid/high SES).
- Influence of older children: Some caregivers reported that their children demand commercial snack foods when they saw older children eating them or if the older child shared them with the younger: “They want to eat junk foods like cheese balls, chips when they see older children eating.” (Mother, working, mid/high SES)/“We share the same social environment with others and the child sees other children having it” (Mother, working, mid/high SES).
- Easy to feed: Caregivers noted commercial snack foods were easy to feed their children: “When we make lito or jaulo we have to coax the child to feed, either play a song or walk around (with them). But for these foods (commercial snack food) we don't have to coax them, they easily eat it” (Mother, working, mid/high SES).
- Alternative when child will not eat anything: Several caregivers reported feeding commercial snack foods when their child refused to eat any other food. Additionally, some noted that when a child was not eating anything, they provided commercial snack foods in the hope it would increase their appetite: “Sometimes (he/she) doesn't eat when I give any food, so I try giving market foods to see if the child eats.” (Mother, low SES)/“Usually the child wants it (chocolate). Instead of keeping him hungry, I give it to my child. It's an option when the child is not eating anything” (Mother, working, mid/high SES).
- To distract or pacify children: Caregivers of all types and SES reported feeding commercial snack foods to their children in order to keep them occupied or pacify them when they were fussy/crying: “Chips are only to distract them when they cry or quarrel.” (Mother, mid/high SES)/“Child troubles (me), that's why we give (juice drinks)” (Grandmother, mid SES).

- Caregivers' perception of commercial snack foods for young children

- Perceived child preference for commercial snack foods and beverages

Snack food and beverage products were perceived as highly liked by young children. Caregivers interpreted their children's preference for certain foods based on facial expressions and if they showed interest in a particular food: “My child does drink the juice, and will finish one whole packet of litchi juice” (Mother, mid/high SES). Commercial packaged foods were consistently ranked highly on the child preference continuum. Savoury snack foods, chocolate, and juice drinks were noted as the foods that were eaten most eagerly by children: “We should place the chocolate at the top, (they) eat it with much pleasure. No matter how much they eat it, they will keep the chocolate in their mouth when given” (Mother, low SES). Many caregivers noted that children preferred commercial foods to home‐made foods and that feeding commercial foods could reduce a child's appetite or willingness to eat home‐made foods: “My child doesn't feel like eating rice if she gets cheese ball, Kurkure (a spicy chip), chocolate, and biscuits.” (Mother, low SES)/“If the child gets these, they would go on eating. Children prefer packaged food over home‐cooked ones” (Mother, working, mid/high SES).

- Perceived unhealthiness and distrust of commercial snack products

Across all caregiver types and SES, there was an agreement that most commercial snack foods and beverages were not considered healthy or nutritious for young children. During ranking exercises, caregivers placed non‐commercial foods—milk, egg, meat, ghee, green leafy vegetables, and fruits—as the most healthy and nutritious, whereas commercial foods—instant noodles, cheeseballs and potato chips, biscuits, juice drinks, and chocolates—were ranked the lowest. Commercial foods were considered to be lacking in nutritional content: “There is no vitamin in it (noodles)” (Mother, low SES). Several caregivers spontaneously used the word “junk food” to describe the nutritional quality of commercial snack foods: “They (juice drinks) have different value, this is junk food” (Mother, working, mid‐high SES).

Caregivers were wary of what they perceived as unhealthy characteristics of commercial snack foods. Ingredients such as monosodium glutamate (MSG) and artificial colouring were considered particularly unhealthy for their children: “Moong dalmot (savory snack) is not that good in my experience because it has MSG.” (Mother, working, mid/high SES)/“They say the seasoning (in noodles) makes the child weak.” (Mother, mid/high SES)/“They say that we should not give much of it (cheeseballs) to children because of the food coloring” (Grandmother, mid SES). Caregivers also reported not trusting some commercially packaged foods because they could not see the product/ingredients and were suspicious of the processing used to manufacture these foods: “They (commercially packaged foods) are seal packed, there are talks that many inedible things were found in these things, and so I don't feel these are healthy” (Mother, working, mid/high SES). Additionally, distrust of manufacturing and expiration dates were reported: “This one (juice) comes in packets, we don't know when it's made” (Mother, working, mid/high SES).

However, caregivers reported trusting several brands of commercial products. Horlicks (a malt powder‐based drink) and Lactogen (a breastmilk substitute) were perceived as healthy because they were fortified and believed to be manufactured in accordance with a child's nutritional requirement: “It (Horlicks drink) is nutritious, helps brain and body development.” (Mother, low/mid SES)/“Milk powder, like Farex and Lactogen, are made according to the child's age, we have to believe these products” (Mother, working, mid/high SES). Caregivers from low/mid SES perceived Horlicks drink as a high‐quality food product that was fed to children by mothers who could afford it.

- Convenience in preparation and feeding of commercial snack foods

Commercial foods were commonly ranked as the most convenient foods to feed as snacks. Caregivers noted that they were easy to prepare/ready‐to‐eat: “Chocolate is very convenient. No need to wash it or slice it” (Mother, low SES). In particular, the combination of milk and biscuits, commonly fed as a breakfast meal for young children, was considered highly convenient across caregiver types and SES groups: “To give biscuits is easy, quite easy, just tear the package and give” (Mother, working, mid/high SES)/“No required cooking, just heat the cold milk and put biscuits, and then feed” (Grandmothers, low/mid SES).

Caregivers' consideration of convenience was not based solely on ease of food preparation but also included if a child ate a food easily and if it was easy to feed. In some cases, foods that required greater preparation time were still considered convenient if a child ate them eagerly/easily, such as *dal bhat*. Conversely, though easy to prepare, *lito* was not considered convenient because children did not like it and they fussed during feeding time. Caregivers ranked commercial foods as convenient because they were easy to feed: “The child eats half of the food (home‐cooked food) and throws away half. They eat such things (commercially foods) themselves, 1‐year‐old child can hold it and eat” (Mother, working, mid/high SES). Convenience of snack foods and beverages, both in terms of reduced preparation time and feeding time, was noted across all groups but was particularly emphasized among working mothers.

- Perceived cost of commercial snack foods

Commercial snack foods were consistently ranked as the least expensive snacks, whereas fruits, meat, dry fruits, and nuts were considered the most expensive. Caregivers noted that commercial products are available in small packages and are not costly when purchased: “They are cheap, yes … These ones (commercial snack foods) can be bought in small packets, and in less amount of money. This one (rice) needs to be bought in more quantity, and less money is not enough” (Mother, working, mid/high SES). Some caregivers felt that costs of commercial food products depended on the quality: “It depends on the quality and size. Some (chocolate) costs 10 rupees, some 20. Small ones are not of good quality.” (Mother, working, mid/high SES)/“If we pay higher, we get better quality” (Mother, working, mid/high SES).

## 4 QUANTITATIVE SURVEY RESULTS

The majority (90.3%, *n* = 673) of survey respondents were mothers, and 7.1% were grandmothers; male caregivers, such as fathers and grandfathers, were rare. The average caregiver age was 29 years, with a range of 17–74 years. Thirteen percent had no formal education, and 14.9% had attended a tertiary level education. Seventeen per cent of primary caregivers reported currently working outside the home, with most of these caregivers involved in sales/service industries. Almost all children (98.4%) had consumed a snack food or beverage product in the week prior to interview; frequency of snack food and beverage consumption in the week prior to interview is presented in Table 1. Biscuits, candy/chocolates, savoury snacks, and instant noodles were the most commonly consumed, having been eaten by 92.1%, 82.8%, 66.0%, and 59.2% of all children, respectively, in the week prior to interview. Juice drinks and malt/chocolate‐based drinks were consumed by approximately one third (38.5%) and one quarter (23.0%) of children, and soft drinks were the least commonly consumed in the week prior to interview (15.3%).

**Table 1:** Frequency of snack food and beverage consumption in previous week (n=745)

Category of snack food/beverage	%(n)
Biscuits	
Every day	41.7 (311)
Most days (4–6 days)	15.6 (116)
Approximately once a week (1–3 days)	34.8 (259)
No consumption in last week	7.9 (59)
Savoury snacks	
Every day	10.1 (75)
Most days (4–6 days)	12.2 (91)
Approximately once a week (1–3 days)	43.8 (326)
No consumption in last week	33.9 (253)
Pastry snacks	
Every day	2.5 (19)
Most days (4–6 days)	3.1 (23)
Approximately once a week (1–3 days)	34.5 (257)
No consumption in last week	59.9 (446)
Candy/chocolate	
Every day	23.6 (176)
Most days (4–6 days)	15.7 (117)
Approximately once a week (1–3 days)	43.5 (324)
No consumption in last week	17.2 (128)
Instant noodles	
Every day	3.0 (22)
Most days (4–6 days)	4.8 (36)
Approximately once a week (1–3 days)	51.4 (383)
No consumption in last week	40.8 (304)
Soft drink	
Every day	0.3 (2)
Most days (4–6 days)	0.8 (6)
Approximately once a week (1–3 days)	14.2 (106)
No consumption in last week	84.7 (631)
Malt/chocolate drinks	
Every day	12.5 (93)
Most days (4–6 days)	3.0 (22)
Approximately once a week (1–3 days)	7.5 (56)
No consumption in last week	77.0 (574)
Juice drinks	
Every day	2.5 (19)
Most days (4–6 days)	4.2 (31)
Approximately once a week (1–3 days)	31.8 (237)
No consumption in last week	61.5 (458)

**Figure 1:**
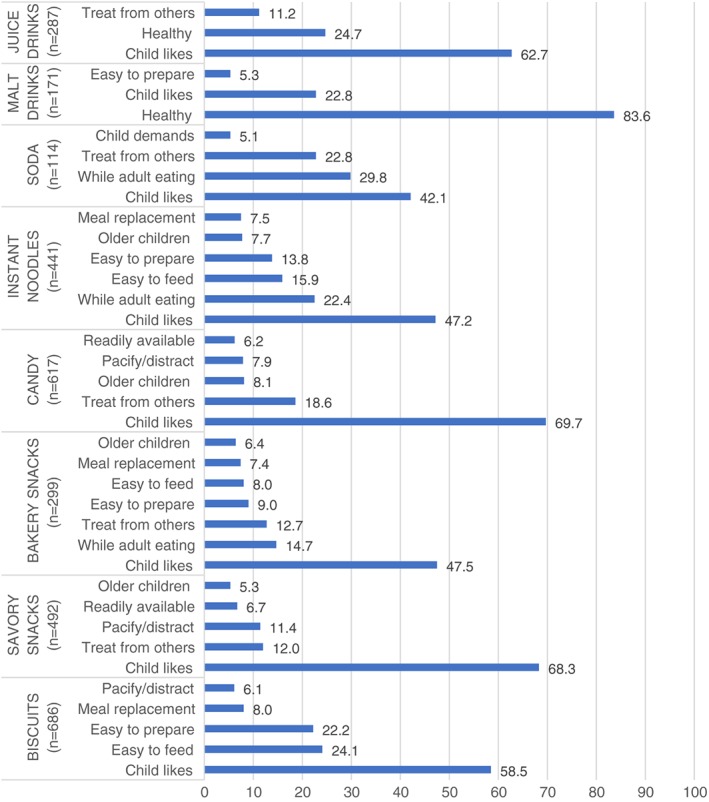
Reported reasons caregivers fed snack foods or beverages, by snack category

Caregivers' reasons for feeding snack food and beverage products to their child 12–23 months of age in the week prior to interview are presented in Figure 1; snack food and beverage products included biscuits/cookies, candy/chocolates, savoury snacks (potato chips, cheeseballs, etc.), bakery snacks (cakes, muffins, donuts, etc.), instant noodles, soda/fizzy drinks, malt/chocolate powder‐based drinks, and juice drinks. Child preference was the most prevalent reason across all categories of snack foods and beverages, except for malt/chocolate powder‐based drinks. Convenience was commonly reported as one reason why caregivers used these foods for young child feeding; half of caregivers who provided a snack food in the previous week (49.3%, n=361) reported doing so because the food was easy to feed or because it was easy to prepare. Biscuits/cookies were the most highly convenient snack food, with nearly a quarter of caregivers who fed biscuits reporting that they fed this food because it was easy to feed (24.1%, n=165) or easy to prepare (22.0%, n=151). Many caregivers who described commercial snack foods or beverages as convenient options because their child eagerly ate them/these foods were easy to feed also reported feeding a snack food because the child liked it (*P* = 0.021). Almost one fifth (18.1%, n=133) of caregivers reported using these foods to pacify or distract an upset child and 15.0% of caregivers fed biscuits/cookies, instant noodles, or bakery snacks as a meal/food alternative for a fussy child who would not eat anything else. Responses that a snack food was fed as a meal replacement/alternative for children who would not eat anything else and responses that these foods were fed to distract or pacify an upset child were also correlated with caregivers who reported feeding these foods because the child liked it (P=0.013 and P=0.002, respectively).

Nearly one third (29.9%, n=219) of caregivers reported feeding snack food or beverage products because they believed they were healthy. This was predominantly driven by snack beverages—83.6% (n=143) of caregivers who fed malt/chocolate powder‐based drinks and 24.7% (n=71) of those who fed juice drinks in the last week reported doing so because they thought it was healthy and/or good for the child's development. The presence/influence of others was commonly reported as a reason why the child ate the snack food or beverage; 32.1% (n=235) of all snacks were fed as a treat from a neighbour or guest, with candy/chocolates the most common snack provided as a treat. Several foods, including instant noodles, soda/soft drinks, and bakery snacks, were commonly fed to the child when either the caregiver or another adult/relative was eating the food themselves. Only seven (1.0%) caregivers who fed snack foods or beverages to their child reported cost as a reason for doing so.

Reasons for using snack foods or beverages were compared between caregiver types and between wealth status groups based on differences noted during FGD. Although grandmothers participating in FGD more commonly reported that snack foods were fed as a treat by other adults, there was no difference in the proportion of surveyed grandmothers reporting that their child ate snack food or beverage because it was fed as a treat, as compared with mothers (25.0% vs. 33.0%; *P* = 0.234). Surveyed grandmothers were more likely than mothers to provide a snack food or beverage to their child as a meal replacement/food alterative when the child would not eat anything else (26.9% vs. 13.9%, *P* = 0.011). Surveyed mothers were more likely than grandmothers to report that their child ate a snack food or beverage because they were influenced by an older child/sibling (18.7% vs. 7.7%, *P* = 0.046). The proportion of caregivers currently working outside the home who reported feeding snack foods or beverages to their child because these foods were convenient in terms of preparation time, compared with caregivers who were not currently working, approached significance (*P* = 0.067). Nearly all (97.1%) of the poorest caregivers who fed a malt/chocolate power‐based beverage to their child in the previous week reported doing so because they believed it was healthy/good for development, as compared with 71.4% of the wealthiest caregivers who fed such a beverage in the previous week (*P* = 0.060).

## 5 DISCUSSION

This study explored caregivers' use of snack food and beverage products for young child feeding, their perceptions of these foods, and reasons for choosing such foods for their children. Feeding of snack food and beverages to young children 12–23 months of age was highly prevalent among caregivers participating in this study, with almost all surveyed children having consumed such a food in the week prior to interview and all FGD generating such foods in free‐listing of snacks fed to children. The driving factors for use of snack food products were child preference, perceived convenience in terms of preparation and feeding, provision of these foods as treats, influence of other household members, and perceived healthiness of certain foods. The low cost of these foods was not found to be a motivating factor.

The influence of child preference on caregivers' use of snack food products for child feeding has been noted widely in literature across geographies (Almeida, Scagliusi, Duran, & Jaime, 2018; Angeles‐Agdeppa, Lana, & Barba, 2003; Babington & Patel, 2008; Feeley et al., 2016; Machín, Giménez, Curutchet, Martínez, and Ares (2016); Kavle et al., 2015; Pries, Huffman, Adhikary, et al., 2016; Pries, Huffman, Mengkheang, et al., 2016; Rahman et al., 2016; Verma & Punia, 2013; Vitta et al., 2016). Child preference was the most common reason reported by caregivers in this study; however, this response may serve as an initial catch‐all response. It is probable that the reason “the child likes it” is coupled with another motivating factor for caregivers, illustrated by the correlations found between this response and responses related to convenience in child feeding and feeding to pacify/distract a child. These correlations in responses may indicate that although caregivers are selecting these foods because of child preference, this preference aids another underlying motivation for the caregiver. Young child feeding that follows children's cues for certain foods, either feeding whatever is easily eaten without fuss or that which a child demands, has been noted in other studies. Chaidez, Townsend, and Kaiser (2011) found that mothers of toddlers in Mexico often followed their child's cues for food preference and fed whatever the child wanted, reporting that they did not want to see the child cry, and mothers in Egypt provided crisps, cakes, and fizzy drinks when a child refused to eat family foods and reported that these foods had a calming effect on fussy children (Kavle et al., 2015).

The use of snack food and beverage products for non‐nutritive feeding (the provision of food for reasons other than health/development, such as feeding for behaviour management) among Kathmandu Valley caregivers is potentially concerning. When used routinely, such approaches to child feeding have been shown to result in diets that are more likely to deviate from dietary recommendations, greater consumption of sugar‐sweetened beverages and processed foods, and reduced consumption of healthier options such as fruits and vegetables (Blaine et al., 2015; Dovey, Staples, Gibson, & Halford, 2008; Kiefner‐Burmeister, Hoffmann, Meers, Koball, & Musher‐Eizenman, 2014; Rhee, 2008). One U.S. study found that when using food to manage a child's behaviour, mothers of preschoolers reported that they paid less attention to the nutritional content of foods (Fisher et al., 2015). Additionally, exposure to certain tastes during infancy and early childhood has been shown to establish preferences (Anzman, Rollins, & Birch, 2010). Many snack food and beverage products are high in sugar or sodium content and formulated to be palatable, thereby potentially setting a path for less healthy dietary preferences throughout life (Birch & Doub, 2014). The mechanisms by which feeding behaviours influence nutritional outcomes in children, not only in terms of food choices by mothers but also the interaction of feeding practices themselves, merit further exploration in LMIC settings. Feeding practices that indulge child demand/preferences can encourage excess energy intake and weight gain among infants (Anzman‐Frasca, Stifter, & Birch, 2012), with several studies showing that “feeding to soothe” can result in inappropriate feeding practices (Stifter, Anzman‐Frasca, Birch, & Voegtline, 2011; Wasser et al., 2011).

The influence of convenience on the use of commercial food and beverage products has been previously noted by caregivers in Nepal (Pries, Huffman, Adhikary, et al., 2016) and South Asia (Prakash, 2015; Rahman et al., 2016). Across both FGD and survey interviews, caregivers commonly reported providing biscuits as a breakfast meal because they were quick to prepare and their children ate them easily, saving time in both tasks. Ultra‐processed foods are manufactured as ready to eat, intentionally highly convenient, and sometimes referred to as “convenience foods” (Botonaki & Mattas, 2010; Brunner, van der Horst, & Siegrist, 2010). However, frequent consumption of such foods has been found to lower the nutritional quality of diets (Louzada et al., 2015a, 2015b; Steele et al., 2016). In this Kathmandu study, working caregivers reported turning to snack food and beverage products in order to save time on food preparation; a study by Verma and Punia (2013) also found that commercial snack foods were preferred among working women because of their perceived convenience and time‐saving attributes. Literature has indicated that women's high workloads may negatively impact child nutrition in Nepal (Cunningham, Ruel, Ferguson, & Uauy, 2015; Malapit, Kadiyala, Quisumbing, Cunningham, & Tyagi, 2015). Further research on use of nutrient‐poor commercial food products among working women would aid further understanding on how this relationship is mediated in a changing food environment.

Family and social context influenced child feeding among caregivers in this study. Children were commonly provided snack food and beverage products when other family members were eating these foods or were provided these foods as a treat from relatives or guests. Similar patterns were found among mothers of toddlers in Mexico, where children were provided tastes of soda or snack foods if another family member was consuming the item and toddlers' older siblings often served as dietary role models (Chaidez Townsend, & Kaiser, 2011). Ventura and Birch (2008) argue that social modelling plays an important and influential role in shaping a child's diet. Conversely, many studies have shown that the influence of social modelling can also improve diet quality among children (Cooke et al., 2004; Fisher, Mictchell, Smiciklas‐Wright, & Birch, 2002; Wardle, Carnell, & Cooke, 2005). This could therefore serve as a mechanism for reducing snack food and beverage product consumption among young children in Nepal. In addition to providing a positive model for consumption, some influential family members, such as grandmothers (Karmacharya, Cunningham, Choufani, & Kadiyala, 2017), could be leveraged to improve feeding practices of other family members. In this current study, grandmothers reported a preference to avoid unhealthy snack foods but were also more likely than mothers to provide these foods to fussy children when other foods were rejected. It is therefore important to not only leverage nutritional knowledge but also ensure confidence among all types of caregivers in their child feeding skills to facilitate nutritious diets.

Discussions with caregivers revealed a tension between perceived negatives of commercial snack foods (unhealthiness), the positives (highly convenient), and their children's preference and demand for these foods. Caregivers repeatedly ranked most snack food and beverage products as “least healthy” and categorized “market foods” as “junk foods,” with the exception of malt‐based beverages. However, despite their stated distrust of packaged foods and knowledge that these foods were of minimal nutritional value, caregivers still provided these foods to their children, noting that convenience or the need to feed a fussy child something appealing outweighed their desire to avoid such foods. Some FGD participants were hesitant to rank these foods highly on child preference because mothers and grandmothers felt they were not good for children. Caregivers reported that they often catered to their child's preferences, even though they considered commercial snack foods to be unhealthy and not nutritious: “Market food has monosodium glutamate, we should not feed them, but sometimes the child doesn't calm down.” (Mother, low SES)/“They (commercial snack foods) are not nutritious, we give these foods when the child gets fussy.” (Mother, low SES)/“The child likes it (savory snack) and I have to give it. The child wants to eat it, prefers it, so I give” (Mother, working, mid/high SES). Although caregivers perceived commercial snack food to be convenient and liked by the child, some discussed that the health and nutrition benefits of foods were more important when selecting snacks for their children: “A packet of cheeseballs can be easy but we have to consider everything. We do consider his choice also. The child's choice, plus nutrition as well is taken into consideration while we feed the child” (Mother, working, mid/high SES).

Similar results have been noted in prior Kathmandu research, where mothers of children 6–23 months of age reported a taboo against feeding packaged foods for fear they would make their children sick; however, nearly all of these mothers provided a commercially produced snack food to their child in the previous week (Pries et al., 2017). In Bangladesh, Rahman et al. (2016) found that mothers perceived packaged snack foods as not safe for children but still reported feeding them because children preferred the taste. Parents often face competing factors when making child feeding decisions, with convenient and flavourful products serving as a reluctant solution (Almeida et al., 2018; Maubach, Hoek, & McCreanor, 2009). If interventions aim to discourage caregivers from relying on unhealthy snack food and beverage products, they could consider integrating features of snack food products that appeal to mothers (palatable to children and easy to prepare/ready to eat) into high‐quality, nutritious food options. It is also important to note that some commercial snacks foods typically high in sugar content, specifically juice drinks and malt/chocolate‐based drinks, were considered healthy by some caregivers. This may indicate that although caregivers generally considered market foods to be unhealthy, advertising/packaging of some products may influence Nepali caregivers' perceptions, as noted by Menger‐Ogle and Graham (2018). Additionally, although caregivers attributed processing characteristics of certain commercial snack foods to be unhealthy, such as additives and colouring agents, no caregivers noted concerns about high sugar content of these foods. Given the rising rates of diabetes in South Asia (NCD Risk Factor Collaboration, 2016) and public health concerns regarding high sugar consumption, there is a need to raise awareness on this issue in Nepal.

There are several limitations to this study. As a cross‐sectional study, it is not possible to ascertain causality of factors influencing caregivers and their feeding behaviours. Although this study indicates that the use of commercial snack foods and beverages is common among Nepali caregivers, further information is needed on the degree that these foods contribute to child diets and how this contribution influences child nutrition. Additionally, this study was limited to an urban location within Nepal, and although urbanization is occurring rapidly, the majority of the national population resides in rural areas. There is a need for further behavioural research into caregivers' demand for these products to test and identify the cause–effect relationships behind such feeding practices. There is also a need to understand factors influencing infant and young child feeding practices of caregivers in rural settings.

To change behaviours, it is necessary to understand the range of factors motivating a behavioural choice. In this study, Kathmandu caregivers were found to hold general knowledge of what is nutritious and not nutritious for their young children but have stated that they need options that are time saving and pleasing to their children and feeding strategies that can address the challenges of young, fussy eaters. Additionally, because social context can influence what children eat, nutritional knowledge among all household members is necessary in order to promote a young child's healthy diet.

## CONFLICTS OF INTEREST

The authors declare that they have no conflicts of interest.

## AUTHOR CONTRIBUTIONS

NS facilitated the focus group discussions and conducted the qualitative data analysis. AP developed the study design, managed data collection, and conducted the quantitative data analysis. NS and AP developed the manuscript. EF, AU, SF, and EZ contributed to study design and provided comprehensive review of the manuscript.
